# Development of small‐molecule based human 20S Proteasome Activators: A novel therapeutic target to combat amyloid plaques in Alzheimer's disease

**DOI:** 10.1002/alz70859_098876

**Published:** 2025-12-25

**Authors:** Poonam Rani, Nimisha Basavaraj, Reddy Peera Kommaddi, Thilagar Pakkirisamy

**Affiliations:** ^1^ Indian Institute of Science, Bangalore, Karnataka India; ^2^ Centre for Brain Research, Bangalore, Karnataka India

## Abstract

**Background:**

Proteostasis, ensuring proteome stability and functionality, relies on components such as the ubiquitin‐proteasome system (UPS) and autophagy. Dysregulation of these pathways, particularly in aging, leads to protein aggregation in neurons—a hallmark of neurodegenerative diseases. Activating proteasome has received most attention, however recent evidence suggests that UPS can clear aggregate proteins and a potential therapeutic target for Alzheimer disease and other protein misfolding diseases.

**Method:**

Phenothiazine‐ and imidazoline‐based small molecules were synthesized and screened in SH‐SY5Y neuroblastoma cells for proteasome activation and cell viability improvement. Docking and molecular dynamics simulations (PDB: 4R3O) showed selective binding to the α1/α2 intersubunit pocket of the proteasome, involving stabilizing interactions such as hydrogen bonding and π‐stacking. Protein activity assays confirmed enhanced 20S proteasome activity. The compounds were highly biocompatible across various cell lines (e.g., HeLa, HEK293T, SH‐SY5Y), with no toxicity even at 200 µM. Pretreatment protected cells from H₂O₂‐induced apoptosis and significantly reduced reactive oxygen species (ROS). In vivo studies in APP/PS1 Alzheimer’s mice evaluated effects on memory, amyloid plaque clearance, and proteasome activity.

**Result:**

Phenothiazine‐ and imidazoline‐based molecules, designed with diverse functional groups and halogen substituents, selectively bound and activated the proteasome via gate‐opening mechanisms. Among tested compounds, one exhibited superior efficacy, enhancing proteasome activity at nanomolar concentrations without toxicity. In APP/PS1 mice, administering compound one (50 mg/kg) for 14 days improved contextual memory, reduced amyloid plaques, and restored proteasome function. In SH‐SY5Y cells, the compound provided neuroprotection against oxidative stress induced by H₂O₂.

**Conclusion:**

This study introduces direct proteasome activation via gate‐opening as a novel therapeutic approach for neurodegenerative diseases. Validated in SH‐SY5Y cells and Alzheimer’s mouse models, the findings demonstrate reduced amyloid plaques, improved memory, and enhanced neuroprotection. These results establish a robust foundation for advancing proteasome activators as therapeutic agents

